# Increase in Central Retinal Edema after Subthreshold Diode Micropulse Laser Treatment of Chronic Central Serous Chorioretinopathy

**DOI:** 10.1155/2015/813414

**Published:** 2015-06-09

**Authors:** Maciej Gawęcki

**Affiliations:** Dobry Wzrok Ophthalmological Clinic, Kliniczna 1B/2, 80-402 Gdańsk, Poland

## Abstract

*Purpose*. Subthreshold diode micropulse laser (SDM) treatment is believed to be safe method of treating clinical entities involving retinal edema. We present a case of serous edematous reaction of the retina to SDM treatment. *Methods*. Case report. *Results*. A patient with chronic central serous chorioretinopathy (CSCR) was treated with SDM Yellow multispot laser. Procedure had been preceded by careful titration of the laser power, which after achieving of the threshold parameter was decreased by 50%. The follow-up visit two days after treatment revealed significant central retinal edema and subretinal fluid. Fundus autofluorescence image showed thermal reaction from the RPE in the form of small spots of hyperfluorescence corresponding to the laser multispot pattern used for treatment. Retinal edema resolved after topical bromfenac and single intravitreal bevacizumab injection. Slight pigmentary reaction from the RPE persisted. *Conclusion*. In the treatment of CSCR, there is a need to significantly reduce threshold SDM power parameters or simply use very low power without titration.

## 1. Introduction

Subthreshold diode micropulse laser (SDM) treatment is a relatively new method of therapy of various retinal disorders, involving retinal edema and/or presence of subretinal fluid [1, 2]. Clinical entities to be treated with SDM include diabetic macular edema (DME), central serous chorioretinopathy (CSCR), and cystoid macular edema (CME) in the course of retinal vein occlusion. The idea of the micropulse laser treatment is to divide the continuous wave of the laser beam into numerous impulses of very short duration. The total added time of the impulses is usually 5 to 15% of the set time of single laser impact. This is called a duty cycle and most commonly set to 5%. Thanks to the presence of the intervals between short impulses, retina cools down and in the result there is no photothermal damage to the tissue. The effect of this laser treatment is strictly metabolic: it stimulates the retinal tissue to produce mediators that have antiangiogenic and antiedematous action. That includes pigment epithelium derived factor (PEDF), beta actin, thrombospondin 1 (TSP1), and other heat-shock proteins. In chronic CSCR, SDM treatment according to numerous studies helps eliminate subretinal fluid and generally promotes the regression of the disease [3–5].

Another issue is using subthreshold technique in laser treatment. It has been advocated by some authors also in classic laser photocoagulation. The idea of subthreshold treatment is to find the threshold power parameter of the laser, when the impact is barely visible on the retina, and then to treat with parameters significantly lower. In SDM treatment, the process of determining threshold parameters is called titration. The time is usually set to 0,2 second and then power gradually increased until impact becomes visible. After that, laser power is reduced to 50 or even 30% of the threshold value and the retina is treated. Authors are not unanimous about the method of titration and the use of titration at all. The first problem is setting the proper spot for titration: is it supposed to be edematous part of the retina or the normal retina? The second is the fact that in the process of titration we actually finally damage the retina. The damage is not significant, but it occurs. Finally arises the question of percentage of reduction of the threshold power of the laser: how much shall we reduce it to still achieve the proper effect? Chong proposes the reduction to 50 to 30% of threshold parameters and titrates on the border of normal and edematous retina [6, 7]. Professor Midena uses SDM treatment for small edemas, lower than 400 *μ*m in central retinal thickness (CRT), and sets the power at fixed 250 mW for 577 Yellow laser [8]. Luttrull advocates using 810 nm laser as it is much safer for the retina and does not use titration method. He sets laser power parameters at 1400 mW based on his empiric experience [9, 10].

Despite these differences, SDM procedure is promoted as being safe with practically no serious complications and no risk of retinal damage. Optical coherence tomography (OCT) and autofluorescence images taken after SDM revealed no sign of any retinal pigment epithelium response, as seen after classic laser photocoagulation.

We would like to present a case of chronic central serous retinopathy with residual retinal thickening that developed sensory retinal detachment after SDM treatment.

## 2. Material and Methods

**Figure 1:**
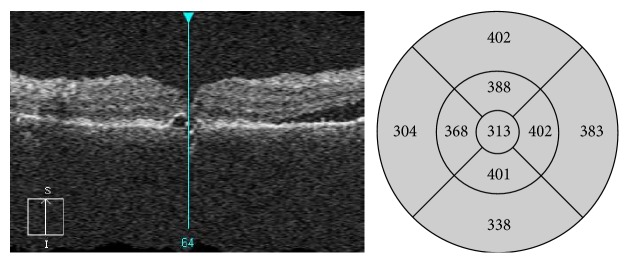
OCT scan of the patient with chronic central serous chorioretinopathy before SDM treatment. General retinal thickening present in the fovea together with small PED. Subretinal fluid present in the superior part of the macula.

**Figure 2:**
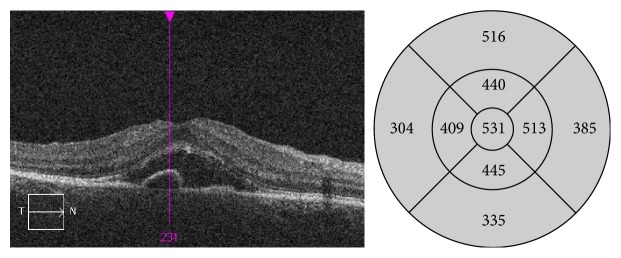
OCT scan 2 days after SDM treatment: large amount of subretinal fluid present as well as persistent small PED. Increase in the thickening of the sensory retina.

**Figure 3:**
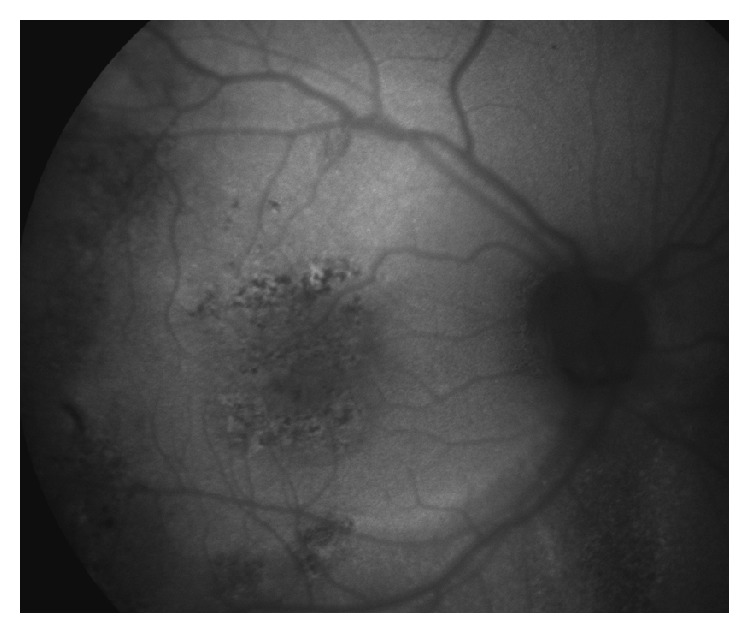
Fundus autofluorescence image taken 2 days after treatment. In the central part, visible alterations of the RPE with pigmentary changes (spots of hypofluorescence) and thermal RPE reaction (spots of hyperfluorescence). On the midperiphery, older retinal changes can be seen, due to previous serous fluid accumulation.

**Figure 4:**
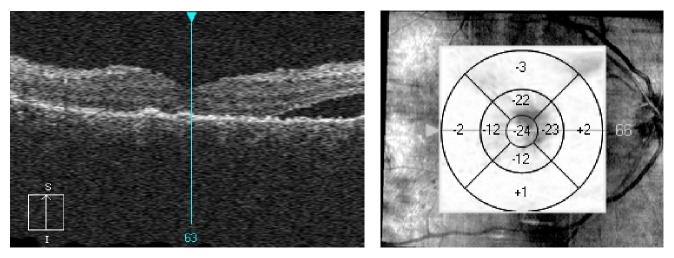
OCT scan a month after SDM treatment. Resorption of the subretinal fluid and slight reduction of the retinal thickening in comparison to the scan taken before laser treatment.

33-year-old female patient was admitted to our outpatient clinic due to chronic serous chorioretinopathy in the RE. LE also suffered from chronic CSCR and in the course of the disease developed epiretinal membrane (ERM) and serious decrease in best corrected visual acuity (BCVA). 5 years ago LE underwent pars plana vitrectomy with ERM peeling and vision stabilized at the level of 0,2. In a few recent years RE had a few episodes of subretinal fluid in the macula. At the time of presentation BCVA in RE was 0,63. On the OCT scan there was retinal thickening present in the foveal region, as well as subretinal fluid outside the foveal area (Figure 1). Patient complained from gradual subjective deterioration of vision in RE and was willing to undergo treatment. As a first line of treatment, intravitreal injection of 1,25 mg of bevacizumab was offered and administered, but it did not bring any effect in terms of reducing retinal thickness or improvement of vision. The patient did not want to continue the treatment because of the cost of injection, so SDM treatment in the macular area was planned. We planned to treat the areas of the retina with subretinal fluid present, as well as the areas of retinal thickening without subretinal fluid. Patient received two sessions of SDM separated by 3-month interval. Treatment was performed with 577 Yellow multispot laser. Before each treatment we employed titration scheme on the edge of edematous and normal retina and reduced laser power by half. We used confluent pattern at the peripheral retina with subretinal fluid and on thickened retina around the foveola, sparing the central part. During one session we made around 600 impacts in the macular region each of 160 *μ*m in diameter, using 550 mW power, 0,2 seconds duration, and 5% duty cycle. After the first session there was no change in central retinal thickness (CRT) and visual acuity. After the second, patient noted significant drop in visual acuity and came back for the follow-up visit in two days. Ophthalmological examination revealed BCVA drop from 0,63 to 0,32. On SD OCT there was significant amount of subretinal fluid present in the macular area (Figure 2). There were no cystoid changes in the sensory retina. Autofluorescence image showed punctate areas of hyperfluorescence referring to multispot laser pattern used for the treatment (Figure 3). Patient was put on topical bromfenac twice a day and was scheduled for anti-VEGF (bevacizumab) treatment in 7 days. One week after the injection significant reduction of amount of subretinal fluid was noted on SD OCT and improvement in BCVA to 0,5. A month after the injection we achieved complete resolution of subretinal fluid. BCVA was back to 0,63—same as before laser treatment—and CRT was slightly reduced as compared to the state before laser therapy (Figure 4). Fundus examination revealed small pigmentary changes corresponding to laser pattern used for treatment.

## 3. Discussion

Presented case report of complication after multispot SDM raises a question of titration method used before this treatment and the extent of the retinal area treated in chronic CSCR. Despite careful titration procedure used in our clinic many times for the treatment of DME and chronic CSCR, we did not avoid overtreatment and in consequence certain retinal damage and retinal shock response in the form of subretinal fluid.

Our titration method was identical to one proposed by Chong. For the titration we chose the spot at the edge of the retinal edema and normal retina, so the power used should have been much lower to the one needed at the edematous retina. The power used for treatment (550 mW) was in fact the average power used in SDM treatment by other authors, for example, Mansour and Gossage for DME [11]. There was also no visible retinal response during treatment itself. The response in the form of pigmentary changes was not visible until the first follow-up visit—2 days after treatment.

The extent of the area of the retina treated by SDM in CSCR also remains a question. Some authors cover the whole area of subretinal fluid presence; others treat only the spots of leakage, based on fluorescein and indocyanine green angiography. Luttrull reports very good results with the SDM treatment of very early diabetic retinopathy with just slight retinal thickening [12]. Based on his extensive experience, we tried to apply his rationale to treat residual retinal thickening in the course of chronic CSCR. We decided to treat the whole area, as our understanding of the SDM is that it stimulates the entire treated retina to faster fluid absorption. In our clinic we spare the central part of the fovea also, which in the context of this case presentation seems to be safer solution.

From our experience we may suggest different approach to setting SDM parameters for treatment of CSCR especially in the foveal region. The power transfer through edematous retina seems to be different in case of cystic changes, typical for DME, and in case of subretinal fluid, or just retinal thickening typical for chronic CSCR. Apparently we overtreated thickened retina, despite no visible reaction during SDM treatment. In cases with alterations of the amount of retinal edema within the treated area, it is difficult to define one fixed power parameter of SDM for the whole area. In cases like that, power titration process may be unreliable and misleading, as it is difficult to find the right spot for titration and simply set power parameters that could be applied to the whole area. We believe that it is safer to skip titration and use safe low power parameters, not exceeding 250–300 mW for 577 Yellow laser. Titration method did not in our case prove to be safe enough to avoid complications.

## 4. Conclusion

SDM treatment in chronic CCSR requires different approach than application of SDM in other clinical entities. In such cases threshold laser power parameters should be significantly reduced after titration (by more than 50%) or even better, simply set at low level of 250–300 mW without titration.
